# Supplementary material for: Geometric Deep Learning for Molecular Crystal Structure Prediction
Source: J Chem Theory Comput. 2023 Apr 13;19(14):4743–56. doi: 10.1021/acs.jctc.3c00031 (PMC10373482; doi:10.1021/acs.jctc.3c00031)
Supplement: Supplementary file 1 — ct3c00031_si_001.pdf [file ct3c00031_si_001.pdf]

Supporting information for "Geometric Deep Learning for Molecular Crystal Structure Prediction"

Michael Kilgour[(1)], *Jutta Rogal*[(1)(2)], *Mark Tuckerman*[(1)(3)(4)(5)]*

[(1)] *Department of Chemistry, New York University, New York, NY 10003, USA*
[(2)] *Fachbereich Physik, Freie Universität Berlin, 14195 Berlin, Germany*
[(3)] *Courant Institute of Mathematical Sciences, New York University, New York, NY 10012, United States*
[(4)] *NYU-ECNU Center for Computational Chemistry at NYU Shanghai, 3663 Zhongshan Rd. North, Shanghai 200062, China*
[(5)] *Simons Center for Computational Physical Chemistry at New York University, New York, NY 10003, USA*

S1. Details of model training

The density regression model used for evaluation in the main text had 4 graph convolution layers and 4 layers in the output MLP, both with a feature depth of 256, totalling 1.97 million parameters. It was trained for 503 epochs, reaching the test minimum at epoch 18. We used the AdamW optimizer on a learning rate schedule starting at 1e-5, increasing exponentially to 1e-4 at epoch 10, and reducing back to saturate at 1e-5 by epoch 60. The batch size was initialized at 10 molecules, (generally with all different numbers of nodes), and increased dynamically to over 4k by the end of training. Ablations undertaken with 1, 8, and 12 convolutional layers, with space group information included in molecule features, and with double the feature depth were undertaken, with very similar results. Similarly, ablation of the input features down to only the atomic number and molecule volume resulted in only slightly worse performance.

The discriminator model was constructed identically to the regression model, except using periodic molecular graph convolution in the last message passing step and with two final outputs instead of one, resulting in 2.08 million parameters. The learning rate schedule was also identical. As discriminator training is much more computationally intensive than for regression, only 50 epochs were completed in a 2 day runtime, saturating the test loss. The batch size was initialized at 88 and dynamically grew to 132 by the end of training. In this case, the initial batch size is set automatically at the beginning of a run to 25% of the maximum batch size the GPU can fit for a certain subset of crystals. This reduction is necessary since molecular graphs sizes may vary widely. The batch size is then allowed to increase incrementally during training until it causes an out of memory error, at which point is reduced again by 40%.

S2. Groupwise submissions analysis for CSD Blind Test 6[1]

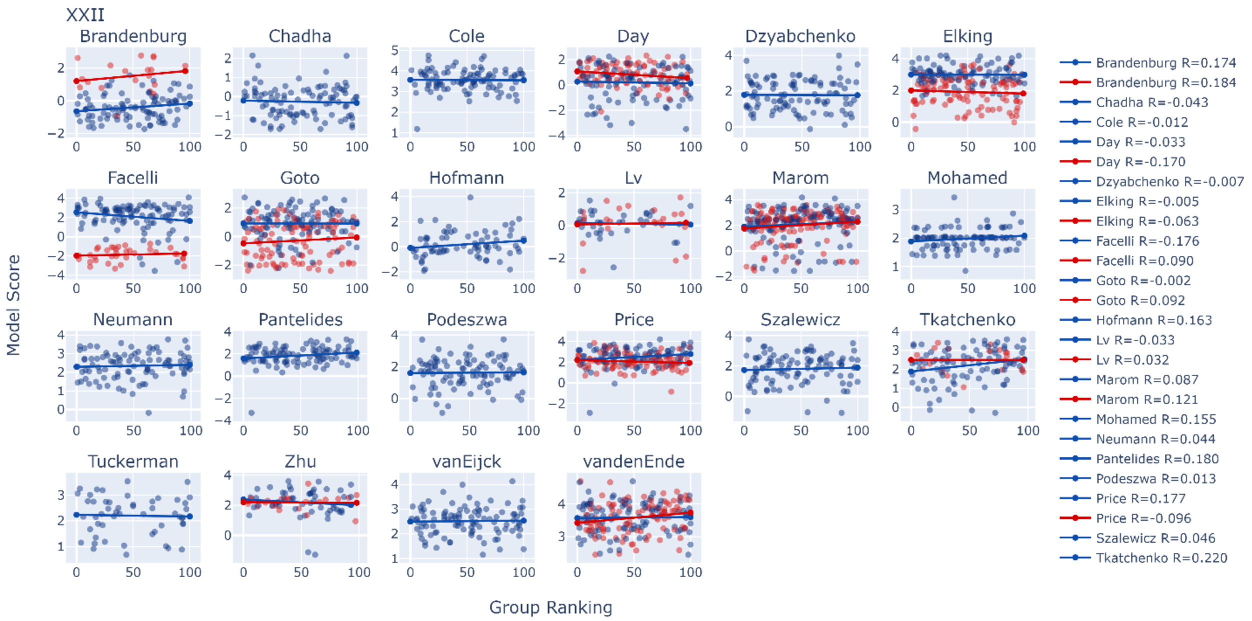

Figure S1: Group ranking vs. model score for CSD Blind Test 6 Target XXII, with lines of best fit and R values.

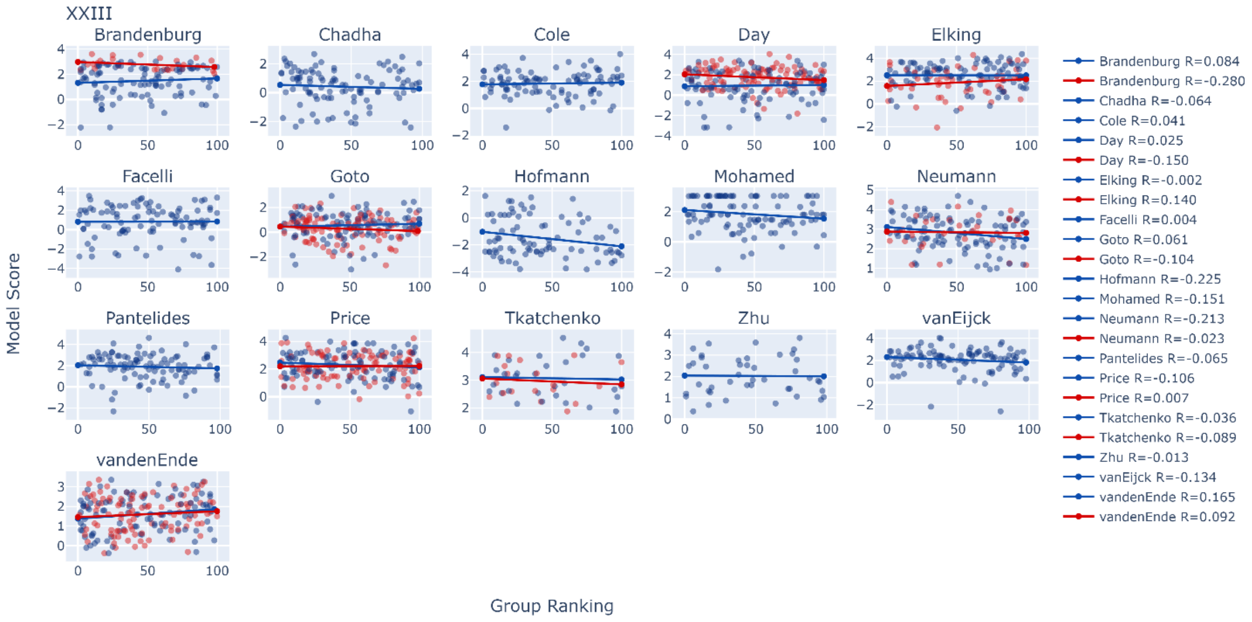

Figure S2: Group ranking vs. model score for CSD Blind Test 6 Target XXIII, with lines of best fit and R values.

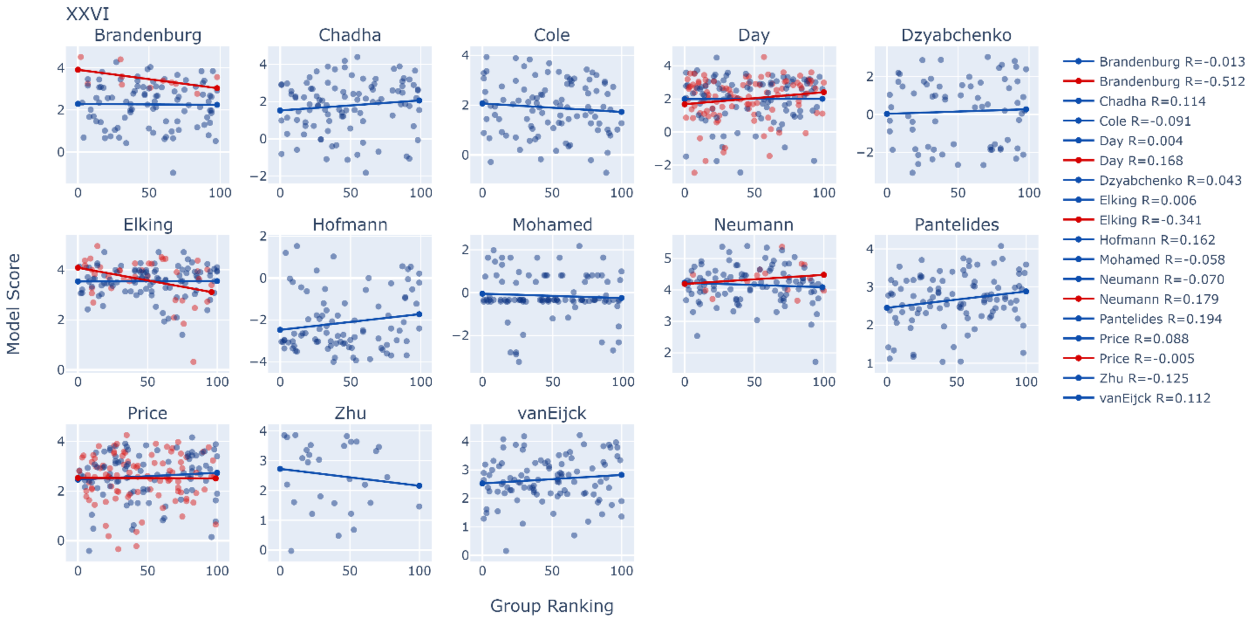

Figure S3: Group ranking vs. model score for CSD Blind Test 6 Target XXIII, with lines of best fit and R values.

S3. Discriminator generalization on various elements & functional groups

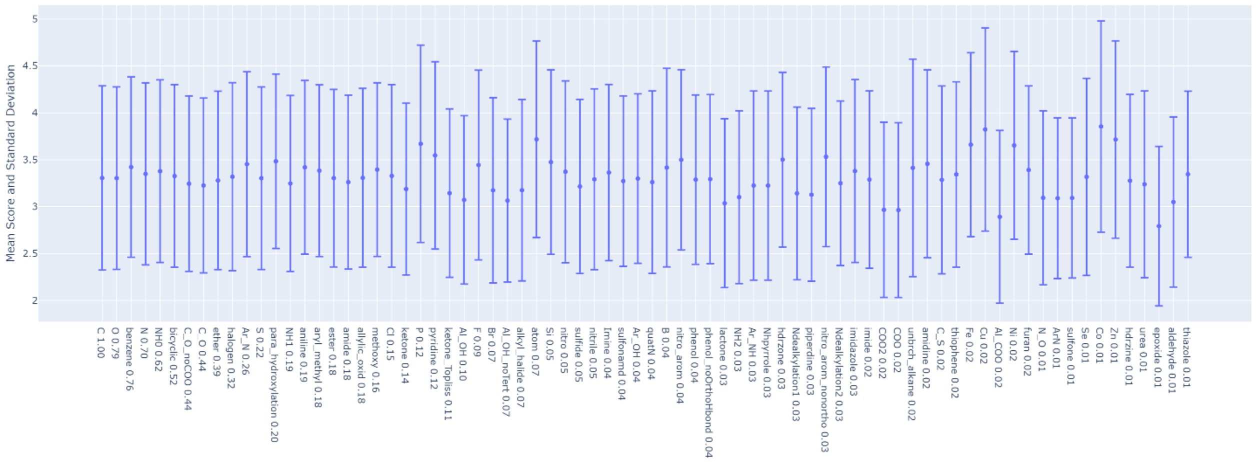

Figure S4: Mean & variance of discriminator scores for samples containing the given elements or RDKit-supplied functional groups, omitting features with less than 1% incidence in the test dataset. Incidence frequency is noted after each label.

References

(1) Reilly, A. M.; Cooper, R. I.; Adjiman, C. S.; Bhattacharya, S.; Boese, A. D.; Brandenburg, J. G.; Bygrave, P. J.; Bylsma, R.; Campbell, J. E.; Car, R.; Case, D. H.; Chadha, R.; Cole, J. C.; Cosburn, K.; Cuppen, H. M.; Curtis, F.; Day, G. M.; DiStasio, R. A.; Dzyabchenko, A.; Van Eijck, B. P.; Elking, D. M.; Van Den Ende,

J. A.; Facelli, J. C.; Ferraro, M. B.; Fusti-Molnar, L.; Gatsiou, C. A.; Gee, T. S.; De Gelder, R.; Ghiringhelli, L. M.; Goto, H.; Grimme, S.; Guo, R.; Hofmann, D. W. M.; Hoja, J.; Hylton, R. K.; Iuzzolino, L.; Jankiewicz, W.; De Jong, D. T.; Kendrick, J.; De Klerk, N. J. J.; Ko, H. Y.; Kuleshova, L. N.; Li, X.; Lohani, S.; Leusen, F. J. J.; Lund, A. M.; Lv, J.; Ma, Y.; Marom, N.; Masunov, A. E.; McCabe, P.; McMahon, D. P.; Meekes, H.; Metz, M. P.; Misquitta, A. J.; Mohamed, S.; Monserrat, B.; Needs, R. J.; Neumann, M. A.; Nyman, J.; Obata, S.; Oberhofer, H.; Oganov, A. R.; Orendt, A. M.; Pagola, G. I.; Pantelides, C. C.; Pickard, C. J.; Podeszwa, R.; Price, L. S.; Price, S. L.; Pulido, A.; Read, M. G.; Reuter, K.; Schneider, E.; Schober, C.; Shields, G. P.; Singh, P.; Sugden, I. J.; Szalewicz, K.; Taylor, C. R.; Tkatchenko, A.; Tuckerman, M. E.; Vacarro, F.; Vasileiadis, M.; Vazquez-Mayagoitia, A.; Vogt, L.; Wang, Y.; Watson, R. E.; De Wijs, G. A.; Yang, J.; Zhu, Q.; Groom, C. R. Report on the Sixth Blind Test of Organic Crystal Structure Prediction Methods. *Acta Crystallogr. Sect. B Struct. Sci. Cryst. Eng. Mater.* **2016**, *72* (4), 439–459. https://doi.org/10.1107/S2052520616007447.
